# Permeability characteristics and structural evolution of compacted loess under different dry densities and wetting-drying cycles

**DOI:** 10.1371/journal.pone.0253508

**Published:** 2021-06-28

**Authors:** Kang-ze Yuan, Wan-kui Ni, Xiang-fei Lü, Xi-jun Wang

**Affiliations:** 1 Department of Geological Engineering, College of Geological Engineering and Surveying and Mapping, Chang’An University, Xi’an, Shaanxi, China; 2 School of Water and Environment, Chang’An University, Xi’an, Shaanxi, China; 3 CCCC First Highway Consultants Co., LTD, Xi’an, Shaanxi, China; China University of Mining and Technology, CHINA

## Abstract

Permeability characteristics of compacted loess is always an important topic in soil mechanics and geotechnical engineering. This study explored the permeability characteristics of compacted loess under different dry densities and wetting-drying cycles, and found that as the dry density increases, the compacted loess surface became denser, the saturation permeability coefficient and saturation infiltration rate decreased. However, the wetting-drying cycle presented the opposite result. Meanwhile, the evolution of the microstructure was investigated by Scanning Electron Microscope (SEM) and Nuclear Magnetic Resonance (NMR) to explain the change of its permeability characteristics. The size of compacted loess aggregates was quantitatively analyzed by Image-Pro Plus (IPP) software. It showed that the size of compacted loess aggregates for different dry densities were concentrated from 10–100 μm, occupying 65.0%, 58.19%, and 51.64% of the total aggregates area respectively. And the interesting finding was that the area occupied by 10–50 μm aggregates remained basically unchanged with the number of wetting-drying cycles increasing. Therefore, the size of 10–50 μm aggregates represented the transition zone of compacted loess. NMR analyses revealed that with increasing dry density, the volume of macropores in the compacted loess rapidly decreased, the volume of mesopores and small pores increased. Meanwhile, the change in micropores was relatively small. The pore volume of the compacted loess after three wetting-drying cycles increased by 8.56%, 8.61%, and 6.15%, respectively. The proportion of macropores in the total pore volume shows the most drastic change. Variations in aggregate size and connection relationships made it easier to form overhead structures between aggregates, and the increased of macropore volume will form more water channels. Therefore, the change in permeability characteristics of compacted loess is determined by aggregate size, loess surface morphology, and the total pore volume occupied by macropores.

## Introduction

Loess deposits cover 10% of the world’s continents, including Asia, Africa, central and southern Europe, the American Midwest, and northern France [1–3]. In northwestern China, loess is extremely important and covers 630,000 km^2^, primarily in the Shanxi, Shaanxi, Gansu, and Ningxia regions. The typical loess is an aeolian deposit with an open and meta-stable structure [4, 5]. Some loess exhibits water sensitivity and tends to settle uncontrollably due to internal structural collapse when wetted [6]. Loess foundation collapse and other geotechnical problems are closely related to water infiltration and permeability characteristics of compacted loess [7]. Therefore, it exploring the permeability characteristics of compacted loess during the water infiltration process is fundamental to understanding loess geotechnical behavior.

Significant researches have been carried out, in order to understand the permeability characteristics of compacted loess foundations. Constant-head saturated permeability test on Q_2_ loess samples were tested by Xu et al. [8], which found that as burial depth increases, saturated permeability decreases, and the pore area is the primary factor controlling loess permeability. Consistent evidences have been provided by Hao et al. [9], which reported that the saturated permeability coefficient of compacted loess has an exponential relationship with initial dry density. Liu et al. [10] conducted a series of air permeability tests on undisturbed loess and remolded loess and showed that for the same water content, the air permeability coefficient during the drying process is always higher than that during the humidification process. Quantitative statistical analysis of the microstructural pore parameters of Malan loess was performed by Li et al. [11], which discussed the relationship between the permeability of loess effect on pore structure and the environmental impact. Raisinghani and Viswanadham [12] exhibited that the permeability of geosynthetic reinforced soil decreases with increasing normal stress. However, current research on the permeability characteristics of loess structure primarily focuses on the first exploration of permeability characteristics. Few studies explaining the reasons for changes in permeability characteristics under different wetting-drying cycles exist. As the number of wetting-drying cycles increases, the compacted loess strength decreases, permeability increases, and deformation increases, causing urban construction projects to undergo deformation and risk instability or damage, which is not conducive to the sustainable city development [13, 14].

In light of these considerations, this work explores the permeability characteristics of compacted loess under different dry densities and wetting-drying cycles. Meanwhile, the microstructural analysis was used to explain the change of permeability characteristics. The size of the aggregates is estimated by IPP software, and aggregates contact relation is observed on the surface of the loess by SEM photomicrographs. The pore size distribution is evaluated via NMR, a non-destructive testing method that ensures that the original microstructure of the compacted loess after different wetting-drying cycles is preserved. The innovative contribution of this study relies on the capacity of providing sound microstructural interpretation for the permeability characteristics of compacted loess with different dry densities and wetting-drying cycles.

## Materials and methods

### Loess samples

**Fig 1:**
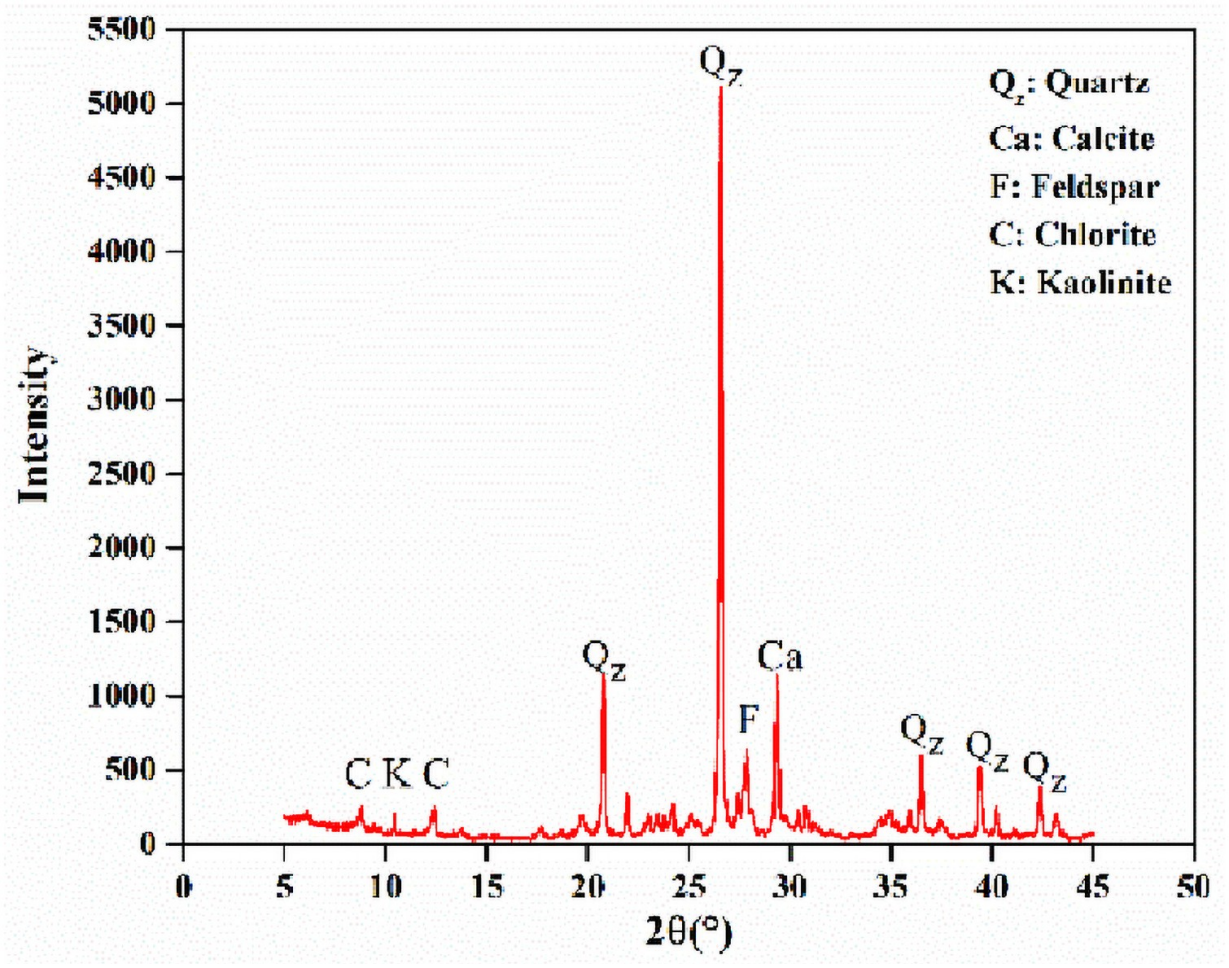
XRD pattern of the original loess sample.

The samples investigated were loess from a foundation pit of loess Plateau in in Yan’an City, China. A total of 85 loess samples (7.0–7.5m depth) with a size of 20×20×40cm were collected in the study area (latitude 35°42′46.09″ N, longitude 109°26′0.10″ E). Loess samples can be classified as Malan loess [15]. All samples were taken on private land. Therefore, please contact Lei Li for future permission. Our sampling sites do not require specific permits and do not involve endangered or protected species. Physical properties of test material were determined following the relevant ASTM standard [16] test methods. According to the XRD pattern of the original loess (Fig 1), the quantitative mineralogical composition was calculated. The underlying physical parameters and mineralogical composition for the loess samples are listed in Table 1.

**Table 1:** Some physical properties of the loess used in this study.

Quantity	value
In situ density(g/cm^3^)	1.35~1.42
Natural moisture content (%)	10
Specific gravity	2.71
Plastic limit (*ω*_*P*_/%)	16.1
Liquid limit (*ω*_*L*_/%)	28.9
Optimal water content w(%)	14.1
Maximum dry density (g/cm^3^)	1.74
Quartz (%)	45.2%
Feldspar (%)	21.0%
Calcite (%)	15.5%
Chlorite (%)	8.0%
Kaolinite (%)	5.8%
Illite (%)	4.5%

### Preparation of loess column

**Fig 2:**
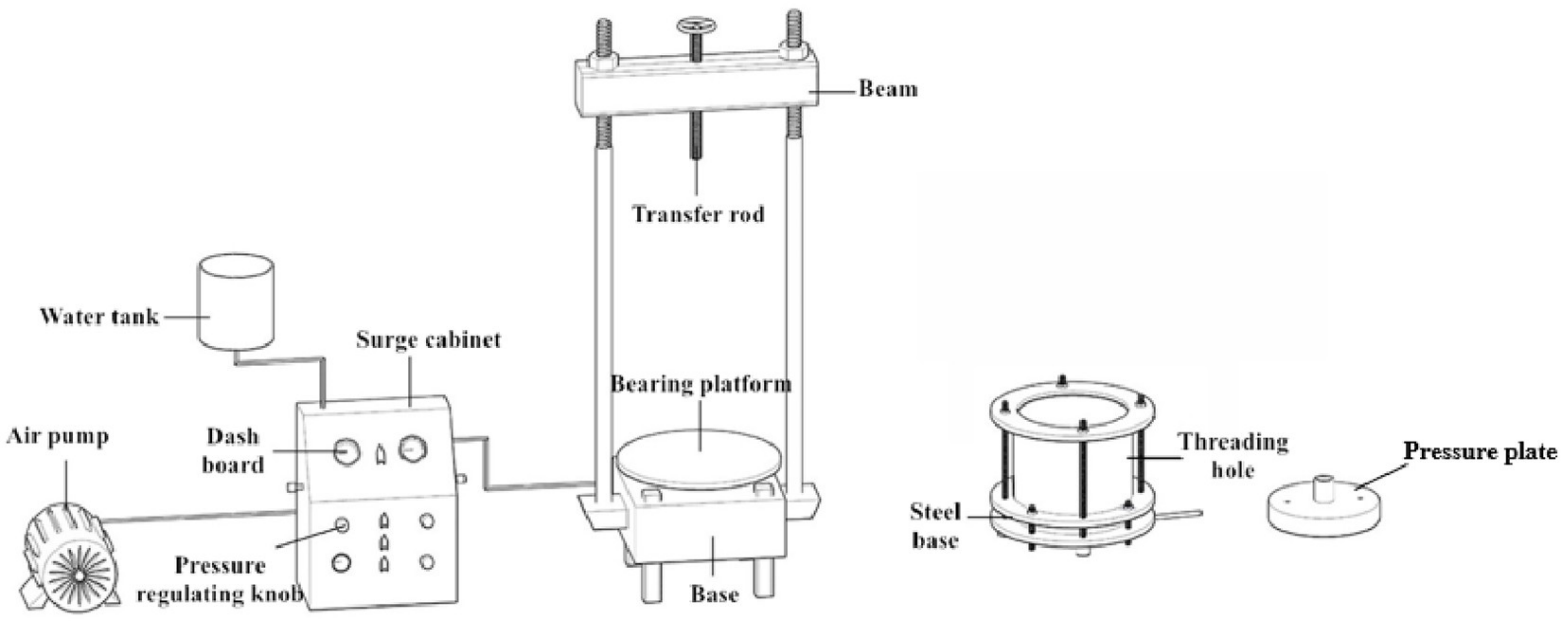
An independently developed device for the large-scale soil column preparation.

The test sample was prepared with an independently developed device for the large-scale soil column preparation (Fig 2). The experimental sample was a loess column with a moisture content of 10%, dry density of 1.45 g/cm^3^, 1.55 g/cm^3^ and 1.65 g/cm^3^, respectively, height of 6 cm and diameter of 30 cm. The preparation process of sample is as follows: The sample preparation process was as follows:

1. Firstly, the original loess was crushed with a wooden hammer until all aggregates were destroyed. These samples were then passed through a 2 mm sieve and then oven-dried at 105°C for eight hours and cooled to room temperature. Thereafter, the amount of deionized water, calculated from Eq 1, was gradually added to the samples using a spray bottle until the moisture content reached the target water content (*w* = 10%). They were tightly sealed with plastic film and placed in a humidor for about 48 hours at room temperature. Part of the processed loess was subjected to a retest of moisture content. If the difference between the average moisture content error after retesting and the target moisture content is within ±0.2%, the sample preparation process can begin. During the processing, the amount of added deionized water was calculated by the Eq 1:
$$m_{w}=\frac{0.01\times \left(w-w_{0}\right)}{1+0.01w_{0}}\times m_{0}$$ (1)
  where *m*_*w*_ is the mass of deionized water added, *m*_*0*_ the mass of the soil sample after drying, *w* is the target moisture content, and *w*_*0*_ is the initial moisture content.
2. Sample preparation was compacted in three layers with each layer being 2 cm high. According to the moisture content, the target dry density and the bottom area of the sample, the required loess quality was calculated for the 2 cm high sample, the surface was smoothed evenly and the horizontal ruler was used to adjust the reaction frame beam;
3. Switching on the air pump and adjusting the pressure control knob to pressurize the loess in stages, with a load of 25 kPa per stage. After the end of the first stage of pressure, the pressure plate was removed, and the height of the soil mass is measured and recorded. When the height of the soil mass after pressure was about 0.5 cm different from the target height (2 cm), the loess mass was slowly pressurized to ensure that the height of the soil mass was accurately maintained at 2 cm;
4. The method of compacting loess in the second and third layers was the same as that of the first compacting loess. After the sample was prepared, let it air-dry to a moisture content of about 1%, and then seal the sample with a film to prevented evaporation of water, and waited for the experiment to proceed.

### Permeability characteristics and wetting-drying cycle

**Fig 3:**
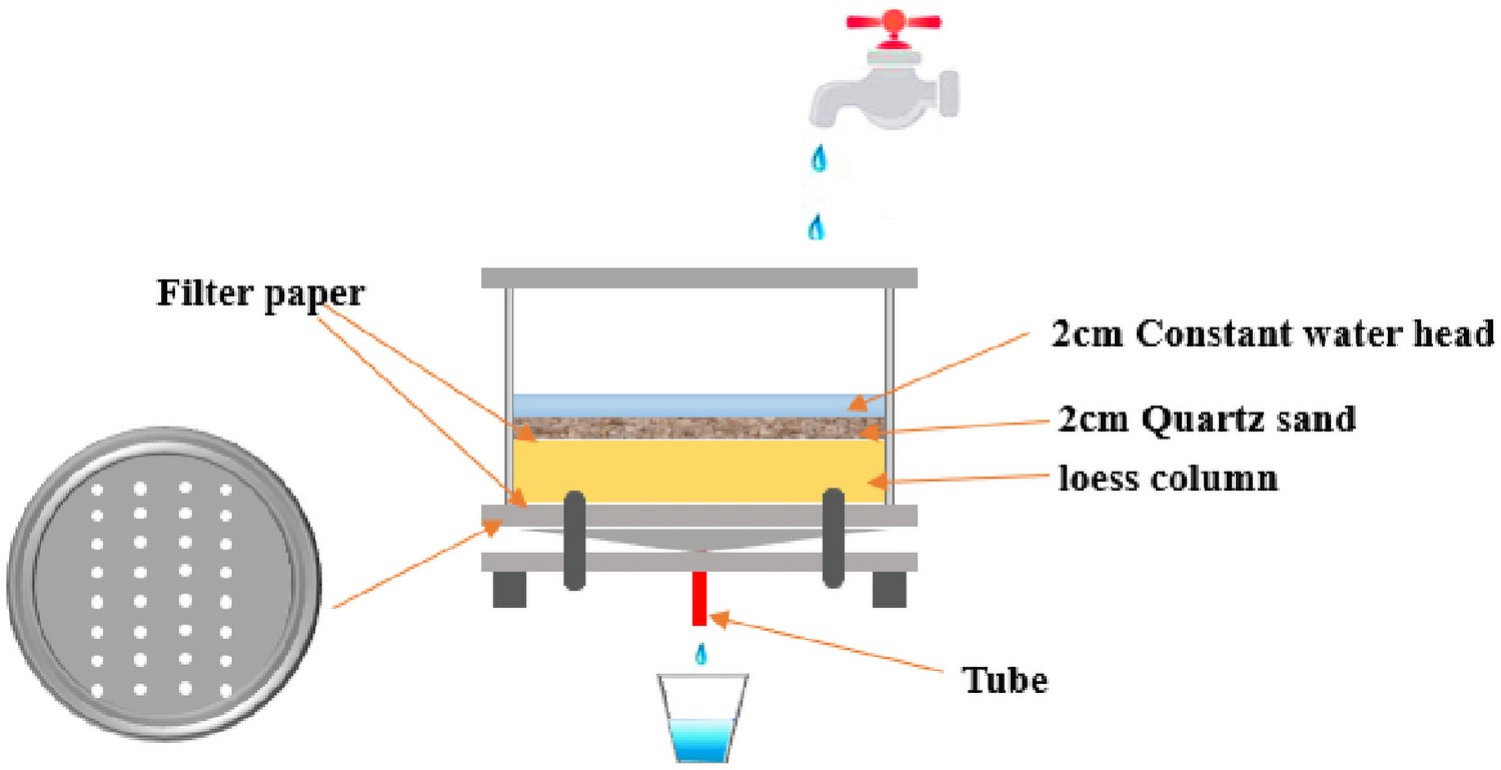
The permeability characteristics test apparatus.

The permeability characteristics statistics and the process of wetting-drying cycles were shown in Fig 3. The bottom tray of the experimental equipment had some small holes, and the presence of filter paper will only allow water to pass through the tube into the container. Furthermore, since temperature is known to affect the permeability of soil greatly [17, 18]. In order to avoid the influence of temperature on *k*_*sat*_, the whole test was done inside constant temperature controller, and the temperature in this study was set to be 22±1°C. After the loess column was prepared with 2.2 sections, 2cm of quartz sand was evenly placed on the top of the loess column to reduce damage to the surface of the loess column. A constant water head of 2cm was strictly maintained on the quartz sand to observe the wetting process of loess column. When the infiltration rate of water in the tube remains stabled for two hour, the wetting process ended, and the loess column was basically in a saturated state. In the wetting process, the infiltration rate was statisticed, and the saturation permeability coefficient was calculated according to Darcy’s law [19] as follows,

$$K_{sat}=\frac{Q}{A∙i∙t}$$ (2)

where *K*_*sat*_ is the saturated hydraulic conductivity (cm/s), *Q* is the volume of water (cm^3^), *A* is the cross-sectional area of the cutting ring (cm^2^), *t* is the time (s) corresponding to *Q*, and *i* is the hydraulic gradient.

In drying process, let the loess column air-dry naturally at room temperature, and tested the weight of the sample. Specimens were weighed regularly until the total mass remained constant for 2 weeks. According to Eq (1), the moisture content of the loess column undergoing the drying process was about 1%. Therefore, the wetting-drying cycle path of loess column was from saturation to water content of 1%. Repeat the above steps until the three wetting-drying cycles test were all completed.

### Microstructure testing

#### SEM

Cubic sticks, with approximately dimensions of 1 cm × 1 cm × 2 cm (length × width × height), were trimmed out from the central part of the loess samples after the wetting-drying cycles were completed. Before scanning, the soil sticks were slightly fractured by hand at about 1 cm height, and the new surface was observed to examine the microstructure of the samples. Then, half the stick was placed on the shooting pad for sputter coating with platinum (Pt) using sputtering ion equipment in conjunction with electron-conductive tape. The fracture plane was not disturbed. A Quanta 200FEG SEM was used to collect the microstructure photos of all samples.

#### Loess aggregate statistics

**Fig 4:**
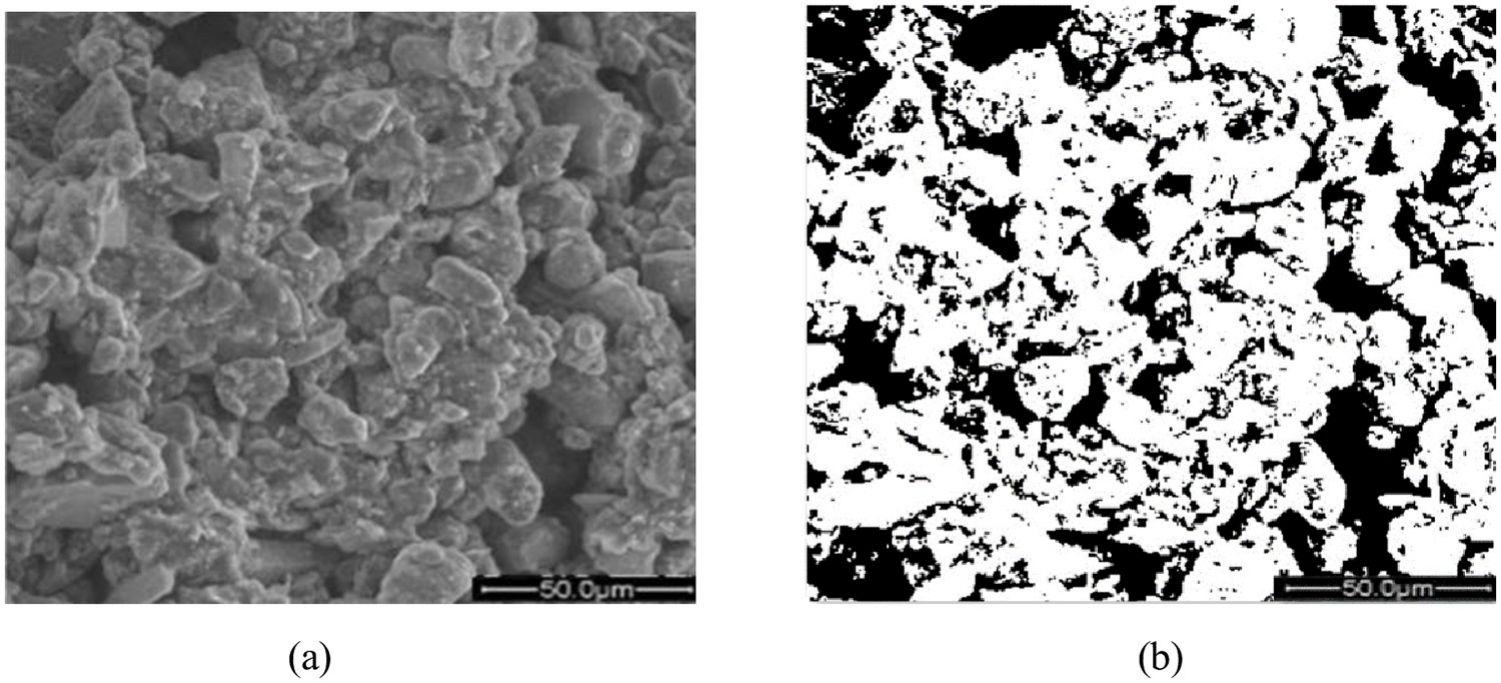
Micrographs of compacted loess. (a) overall view; (b) binarized view (white-colored areas are particles, and black areas are pores).

In order to quantify the aggregate size in the loess structure, IPP image software was used to distinguish the loess particles and pores. SEM micrographs, magnified 1000-fold, were processed to observe the particle distribution in the loess structure. Quantitative processing was carried out as follows. First, the micrographs were imported into the IPP software as binarized images (Fig 4). By examining the binarized images, loess aggregates and pores were easily separated from the soil. The image threshold was the core of binarization. We varied the grey value of each image and captured images three times to calculate the standard deviation.

#### Theoretical background of NMR

**Fig 5:**
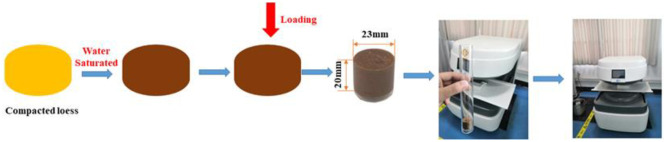
Preparation and test process for NMR sample.

Geophysical NMR methods were used to analyze the pore size distribution in loess samples using MacroMR12-150H-1. Compacted loess samples from the cutting ring were saturated under vacuum conditions and fixed in a specially made quartz tube (Ø 23 mm, height 20 mm). Then, the quartz tube containing the loess sample was placed in the testing tube to obtain a T_2_ curve. This process is shown in Fig 5.

For water-saturated loess samples, T_2_ can be obtained from Eq (3) based on the NMR relaxation mechanisms:

$$\frac{1}{T_{2}}=\frac{1}{T_{2B}}+\frac{1}{T_{2S}}+\frac{1}{T_{2D}}$$ (3)

where, *T*_*2B*_ is the bulk water relaxation time, T_*2S*_ is the surface-enhanced relaxation time on the pore walls, and *T*_*2D*_ is the diffusion relaxation time that accounts for the transverse relaxation in an inhomogeneous magnetic field. For water, *T*_*2B*_ is much larger than *T*_*2S*_ and *T*_*2D*_, so the effect of *T*_*2B*_ on *T*_*2*_ can be neglected. In fact, for pore water in porous loess, the T_*2*_ of the pore water in the soil is directly related to the internal structure of the loess pores:

$$\frac{1}{T_{2}}=\frac{1}{T_{2S}}=\rho \frac{S}{V}=\rho \frac{\alpha }{r}$$ (4)

where, ρ (μm/s) is the surface relaxivity coefficient characterizing the magnetic interactions at the water-loess particles interface, and *S/V* (m^-1^) is the ratio of the pore surface area (*S*) to the pore water volume (*V*). The *S/V* ratio is proportional to the reciprocal pore radius (*r*), expressed as *$\frac{\alpha }{r}$*. In Eq (4), the geometry factor (*α*) depends on the pore shape (*α* = 1 for planar, *α* = 2 for cylindrical, *α* = 3 for spherical shape). In this study, we assume that the pores in loess samples are cylindrical. Therefore, for cylindrical pores, Eq (4) can be written as follows:

$$\frac{1}{T_{2}}=\rho \frac{\alpha }{r}=\rho \frac{2}{r}$$ (5)

and it can be simplified to Eq (6):

$$T_{2}=\frac{1}{2\rho }r$$ (6)

From Eq (6), the relaxation time (T_2_) shows a linear relationship with the pore radius (*r*). Thus, if we know the value of the surface relaxivity coefficient (*ρ*), the pore size distribution in loess samples can be obtained by measuring the T_2_ distribution.

To determine *ρ*, the widely accepted NMR-permeability equation, known as the Schlumberger-Doll Research (SDR) equation developed by Kleinberg et al. [20], is used to obtain *ρ*:

$$K_{s}=C\emptyset^{4}T_{2LM}^{2}$$ (7)

According to Kleinberg et al. [21], the constant (C) of the SDR equation is expected to be proportional to the square of *ρ*. Therefore, the equation is as follows:

$$K_{s}=\rho^{2}\emptyset^{4}T_{2LM}^{2}$$ (8)

and it can be also written as:

$$\rho =\sqrt{\frac{k_{s}}{\emptyset^{4}T_{2LM}^{2}}}$$ (9)

where, *K*_*s*_ is the saturated soil permeability, ∅ is the saturated porosity of the NMR samples, and *T*_2*LM*_ is the geometric mean of the T_2_ distribution.

Among them, a dry density sample of 1.45 g/cm^3^ was taken as an example. The saturated permeability of loess (*K*_*s*_) is 1.12 × 10^−13^ m^2^, the saturated porosity of the soil (∅) is 0.501, the mean geometric value of the T_2_ distribution is 0.52989 ms. Inserting it in Eq (9), the value *ρ*_2_ of 2.69 um/ms is obtained. In the same way, *ρ*_2_ values of dry density of 1.55 kg/m^3^ and 1.65 kg/m^3^ can be calculated as 2.51 um/ms and 2.25 um/ms, respectively.

## Result and discussion

### Statistics of permeability characteristics

**Fig 6:**
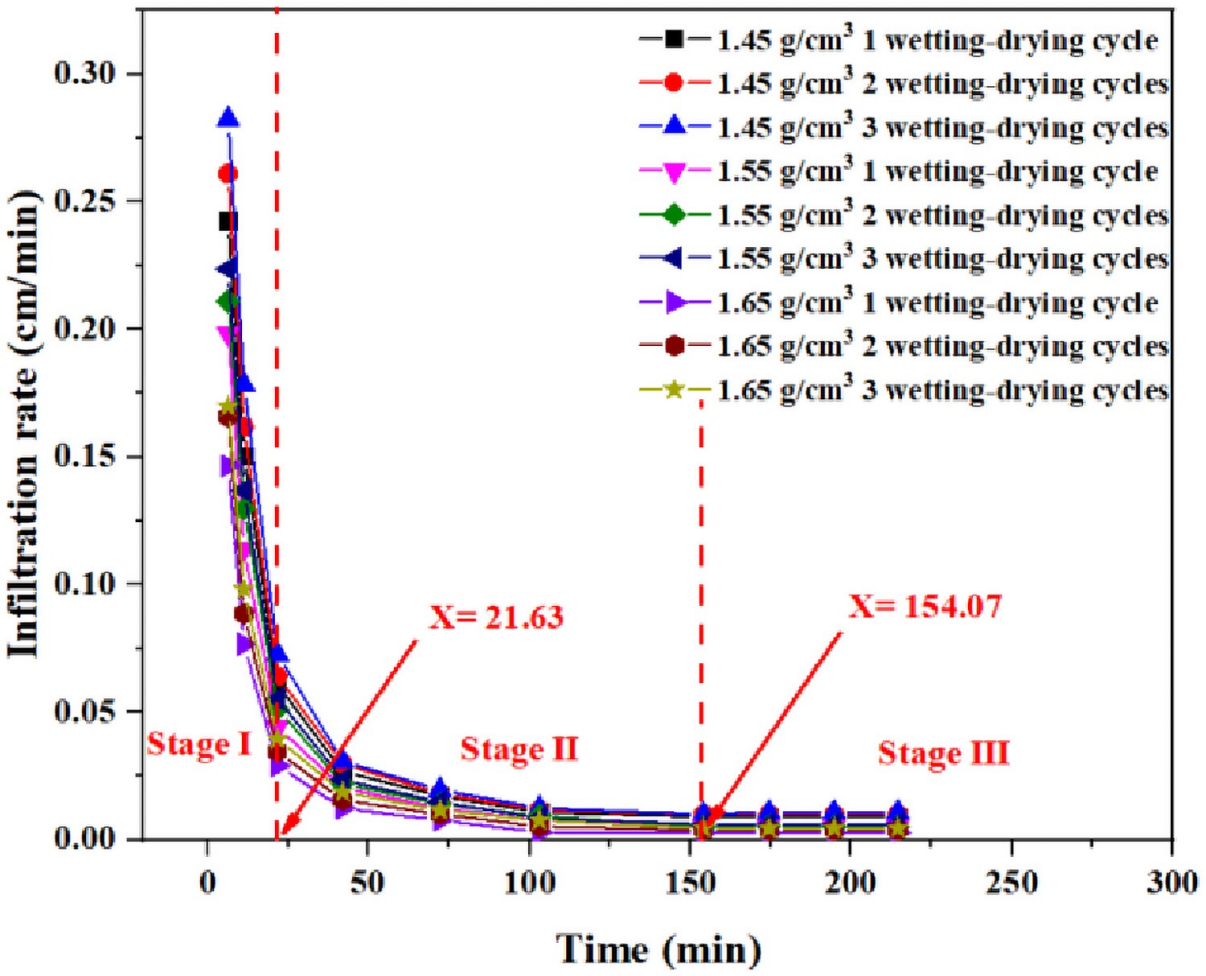
The relationship between infiltration rate and time during wetting-drying cycles.

The relationship between the infiltration rate and time during the wetting-drying cycles of the compacted loess with different dry densities is shown in Fig 6. The infiltration rate of all the wetting-drying cycles of the three groups of compacted loess showed a rapid decrease with increasing time and eventually stabilized. The variation in infiltration rate can be roughly divided into three stages. The stage (I) occured at the initial stage of infiltration (0–21.63 min). The loess column had strong infiltration capacity and the infiltration rate varies significantly. Infiltration rate reached the maximum value at the beginning of the infiltration, and the infiltration rate showed a linear downward trend with increasing time. The second (II) stage was between 21.63 min and 154.07 min, that is, the time from the initial stage of infiltration to the bottom of the water. During this time period, the infiltration rate gradually decreased with increasing time. The stage (III) was after the completion of water infiltration. At this time, the loess column was completely wetted and close to saturation. The infiltration rate hardly changed and is basically stable, similar to a stable seepage state. The loess column infiltration rate with a dry density of 1.45 g/cm^3^, 1.55 g/cm^3^, and 1.65 g/cm^3^ finally stabilized at 8.94×10^−3^ cm/min, 4.95×10^−3^ cm/min, and 2.63×10^−3^ cm/min, respectively. Therefore, within the same wetting-drying cycle, the higher the dry density, the lower the saturated infiltration rate and the more difficult the water infiltration. Under the same dry density, as the number of wetting-drying cycles increases, the saturated and initial infiltration rate increases.

**Fig 7:**
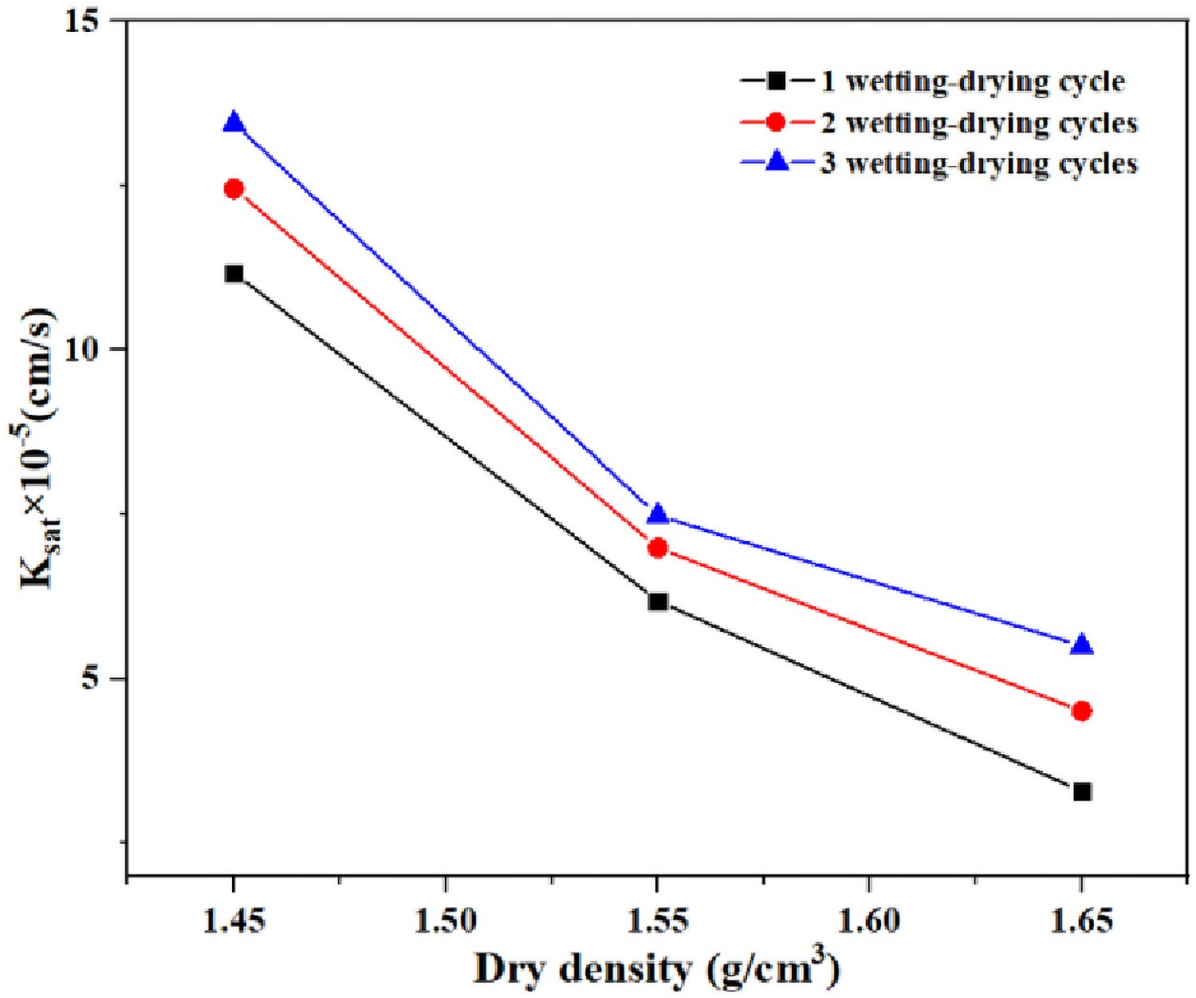
Variation of saturation permeability coefficient (*K*_*sat*_).

According the Eq (2), the saturation permeability coefficient (*K*_*sat*_) can be obtained (Fig 7). It can be seen that the saturation permeability coefficient decreases with the increase of dry density. However, the wetting-drying cycle presented the opposite result. With the increase of wetting-drying cycles, the *K*_*sat*_ of the compacted loess with dry density of 1.45 g/cm^3^ increases rate by 11.45% and 20.33%, respectively. For compacted loess with dry density of 1.55 g/cm^3^, the increase of *K*_*sat*_ from the initial value is 13.04% and 21.01%. As for compacted loess with dry density of 1.65 g/cm^3^, the relative increase of *K*_*sat*_ is 37.12% and 67.33%. The higher the dry density, the higher the saturation coefficient increases rate in the corresponding wetting-drying cycles, it indicates that the wetting-drying cycles have the most obvious effect on the structure of high-density compacted loess.

### Morphology of loess after wetting-drying cycles

**Fig 8:**
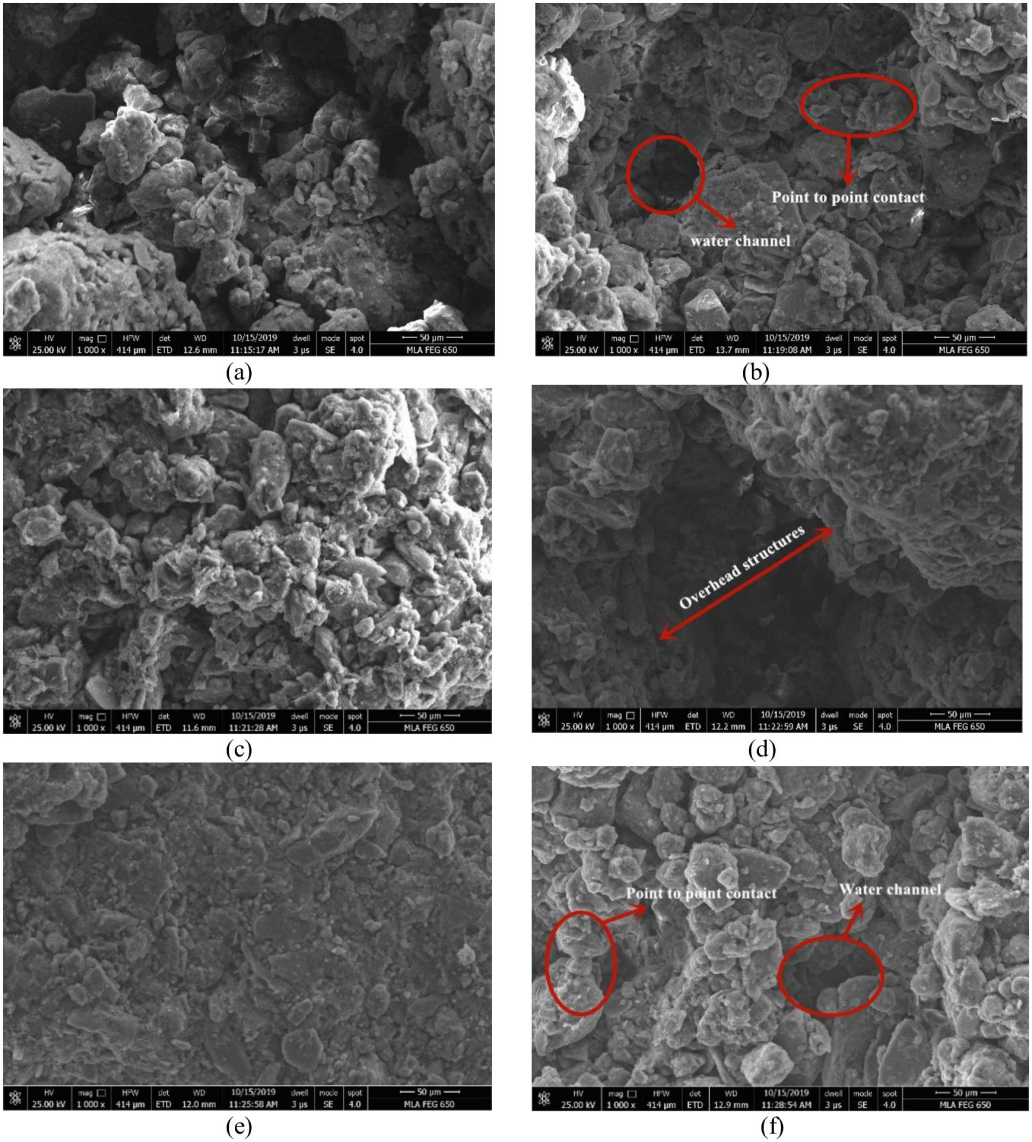
The surface morphology of compacted loess before and after wetting-drying cycles. (a: 1.45 g/cm^3^ 0 time; b: 1.45 g/cm^3^ 3 times; c: 1.55 g/cm^3^ 0 time; d: 1.55 g/cm^3^ 3 times; e: 1.65 g/cm^3^ 0 time; f: 1.65 g/cm^3^ 3 times).

The morphology of the skeleton particles is compared at 1000 times magnification for each dry density before and after wetting-drying (Fig 8). Fig 8a shows the surface morphology with a dry density of 1.45 g/cm^3^. The particles form an overhead structure with notable large pores. For compacted loess with a dry density of 1.55 g/cm^3^ (Fig 8c), the contact between the aggregates in the loess is closer, and the macropores and mesopores in the loess structure are significantly reduced. For compacted loess with a dry density of 1.65 g/cm^3^ (Fig 8e), the compacted loess become more densely and presented a layered structure. When the dry density is small, more overhead structures are present. However, with increasing dry density, the overhead structure gradually disappears, and the mosaic structure becomes the primary structure, which is more compact. The connection relationship of “face to face” contact increase, and the skeleton strength increased, resulting in the loess structure becoming more robust. After three wetting-drying cycles, the loess structure loosens, and the reduction of cementation material lead to increasing amounts of macropores and mesopores within the loess structure. More visible "water channel" presents on the microscopic image of the loess after the wetting-drying cycles, which is the water infiltration path during the wetting process. The wetting-drying cycles process dissolves and scours the cementing materials between aggregates, which weakens the cementation inside the aggregates and turns a large aggregate into a number of particles or aggregates of different sizes, which increases the number of medium and small aggregates as well as weakens the overall skeleton strength.

### Quantitative analysis of aggregate size

Microscopic images of 1.45 g/cm^3^, 1.55g/cm^3^, 1.65 g/cm^3^ compacted loess before and after the wetting-drying cycles were used in the IPP software to calculate the aggregate size and the total area ratio of the various aggregate sizes (Table 2). The size of compacted loess aggregates with different dry densities are concentrated from 10–100 μm, occupying 65.0%, 58.19%, and 51.64% of the total particle area respectively [22]. As dry density increases, the proportion of aggregates smaller than 2 μm in total area increase from 1.06% to 3.06%. The proportion of aggregates with a diameter of 2–5 μm in total area increase from 6.21% to 15.63%, and the proportion of aggregates with a diameter of 5–10 μm in total area increase from 11.21% to 18.21%. When the aggregate diameter is greater than 10 μm, the proportion of the total area occupied by the aggregates gradually decreases as dry density increases. The proportion of aggregates with a diameter from 10–50 μm decreased from 31.58% to 26.32%. The proportion of the total area occupied by aggregates with a diameter of 50–100 μm decrease from 33.42% to 25.23%. When the aggregate diameter is greater than 100 μm, the proportion of the total area decreases from 16.52% to 11.55%. As the number of wetting-drying cycles increases, the total area of aggregates larger than 50 μm gradually decreases, and the total area of aggregates less than 10 μm gradually increases. However, the area occupied by 10–50 μm aggregates remains basically unchanged, proving that the number of aggregates with a diameter from 10–50 μm increase with increasing wetting-drying cycles, and the rate of decrease and increase of the aggregates is similar. Therefore, aggregates from 10–50 μm in diameter represent the wetting-drying cycle transition zone of compacted loess.

**Table 2:** Variation of the total area of different aggregate sizes proportion.

Dry density (g/cm^3^)	Wetting-drying cycles	Proportion of total area occupied by different aggregate sizes (%)					
		< 2 μm	2–5 μm	5–10 μm	10–50 μm	50–100 μm	> 100 μm
1.45	0	1.06	6.21	11.21	31.58	33.42	16.52
	3	2.60	8.91	13.21	30.88	29.89	14.52
1.55	0	2.54	11.37	14.58	27.22	30.97	13.32
	3	2.98	13.91	17.28	27.72	27.36	10.15
1.65	0	3.06	15.63	18.21	26.32	25.23	11.55
	3	3.12	16.15	20.85	26.11	22.93	10.84

### NMR T_2_ test results

**Fig 9:**
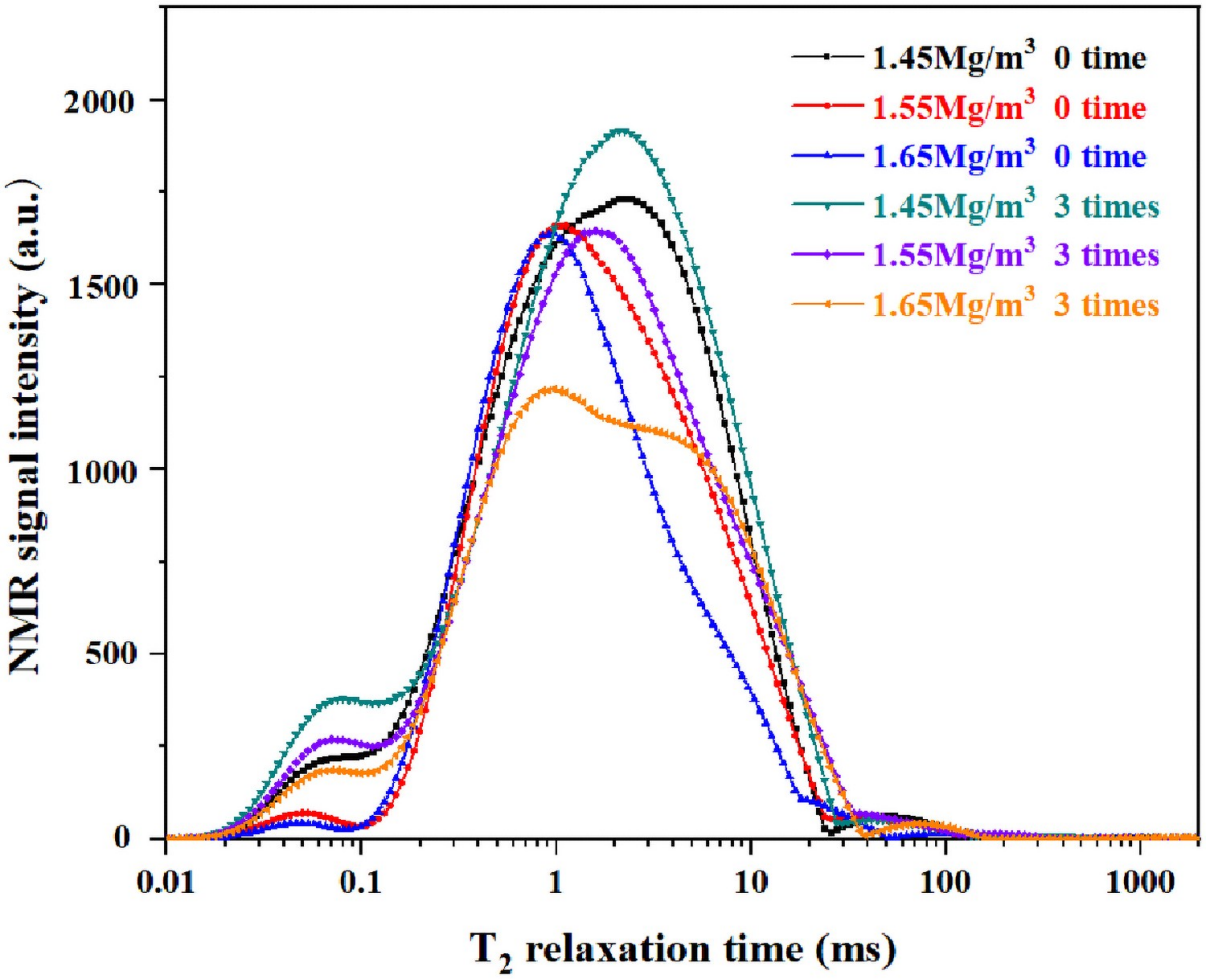
T_2_ distribution curves for different dry densities.

**Fig 10:**
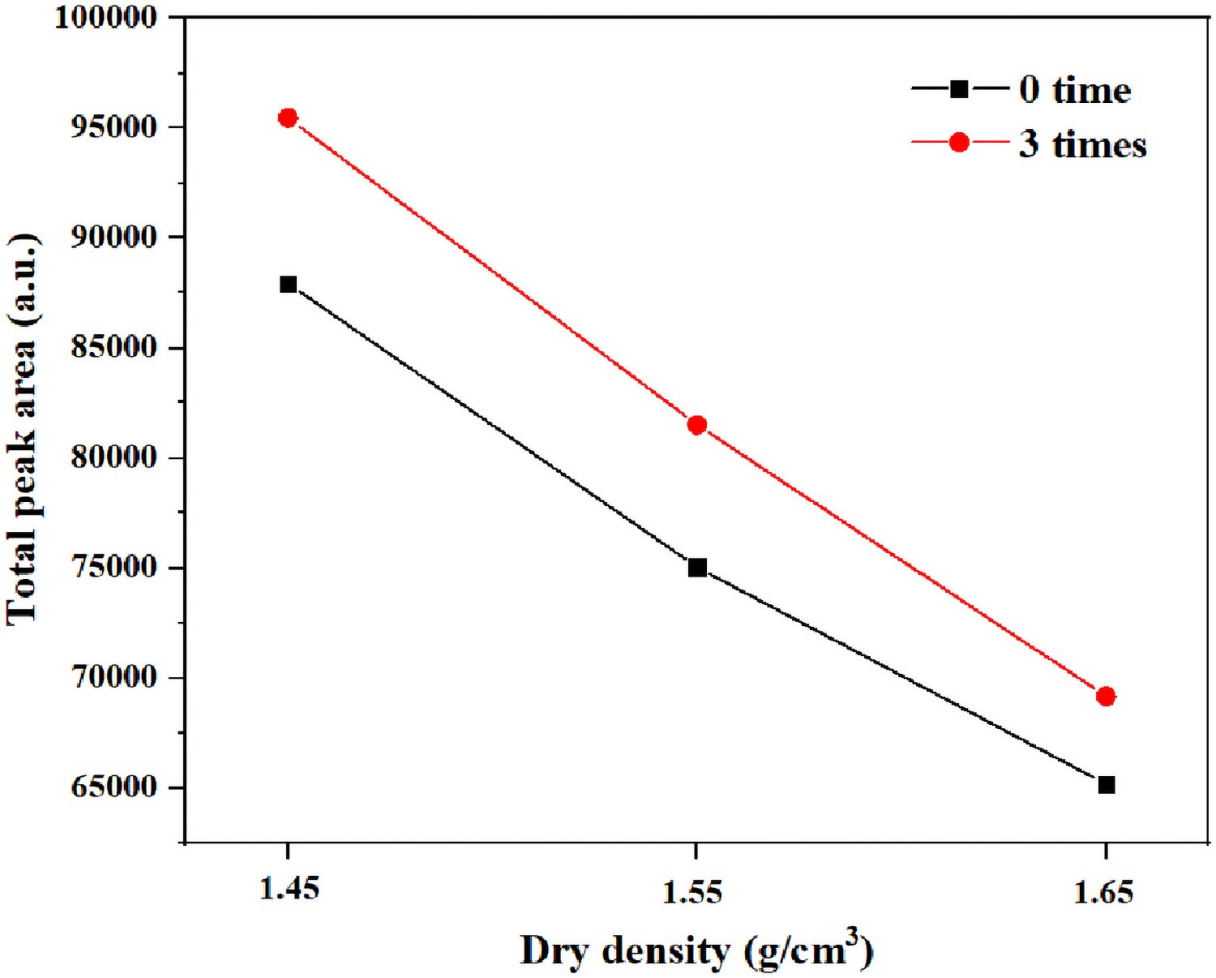
Variation of peak area.

The relaxation time distribution is normalized to the sum of all amplitudes. Each amplitude then represents the proportion of water corresponding to the water relaxation time (the decay of the NMR signal) [23]. The distribution curve of transverse relaxation times T_2_ of the wetting-drying cycle samples under different dry densities in the NMR experiment shows a trimodal relaxation distribution (Fig 9). While short relaxation times correspond to small pores, long relaxation times correspond to large pores. The trimodal relaxation distribution indicates that macro-, meso-, and micropores were present in all loess samples after wetting-drying. The comparison of NMR T_2_ distribution curves shows that the NMR signal proportion decreases with increasing wetting-drying cycles. The comparison of the NMR T_2_ distribution curves results shows that the proportion of NMR signals gradually decreases with increasing dry density, but the proportion of NMR signals increases with increasing number of wetting-drying cycles. The variations in total peak area relative to dry density and the number of wetting-drying cycles are shown in Fig 10. The dry density increased from 1.45 g/cm^3^ to 1.55 g/cm^3^. The total peak area decreased from 87934.5 ms to 75034.9 ms, a 14.7% decrease. When the dry density increased from 1.55 g/cm^3^ to 1.65 g/cm^3^, total peak area decreased from 75034.9 ms to 65162.5 ms, a decrease of 13.2%. For the same dry density, the total peak area increased after three wetting-drying cycles. For compacted loess with a dry density of 1.45 g/cm^3^, the total peak area increased from 87934.5 ms to 95464.4 ms, an increase of 8.56%. The total peak area of the compressed loess with dry density of 1.55 g/cm^3^ increased by 8.61% from 75034.9 ms to 81499.9 ms. For the compacted loess with dry density of 1.65 g/cm^3^, the total peak area increased from 65162.5 ms to 69167.8 ms, an increase of 6.15%. Because the signal amplitude of the T_2_ distribution curve representing the pore water content. Therefore, the volume of the pores in the compacted loess after three wetting-drying cycles increases by 8.56%, 8.61%, and 6.15%, respectively.

### Pore size distribution of loess samples

**Fig 11:**
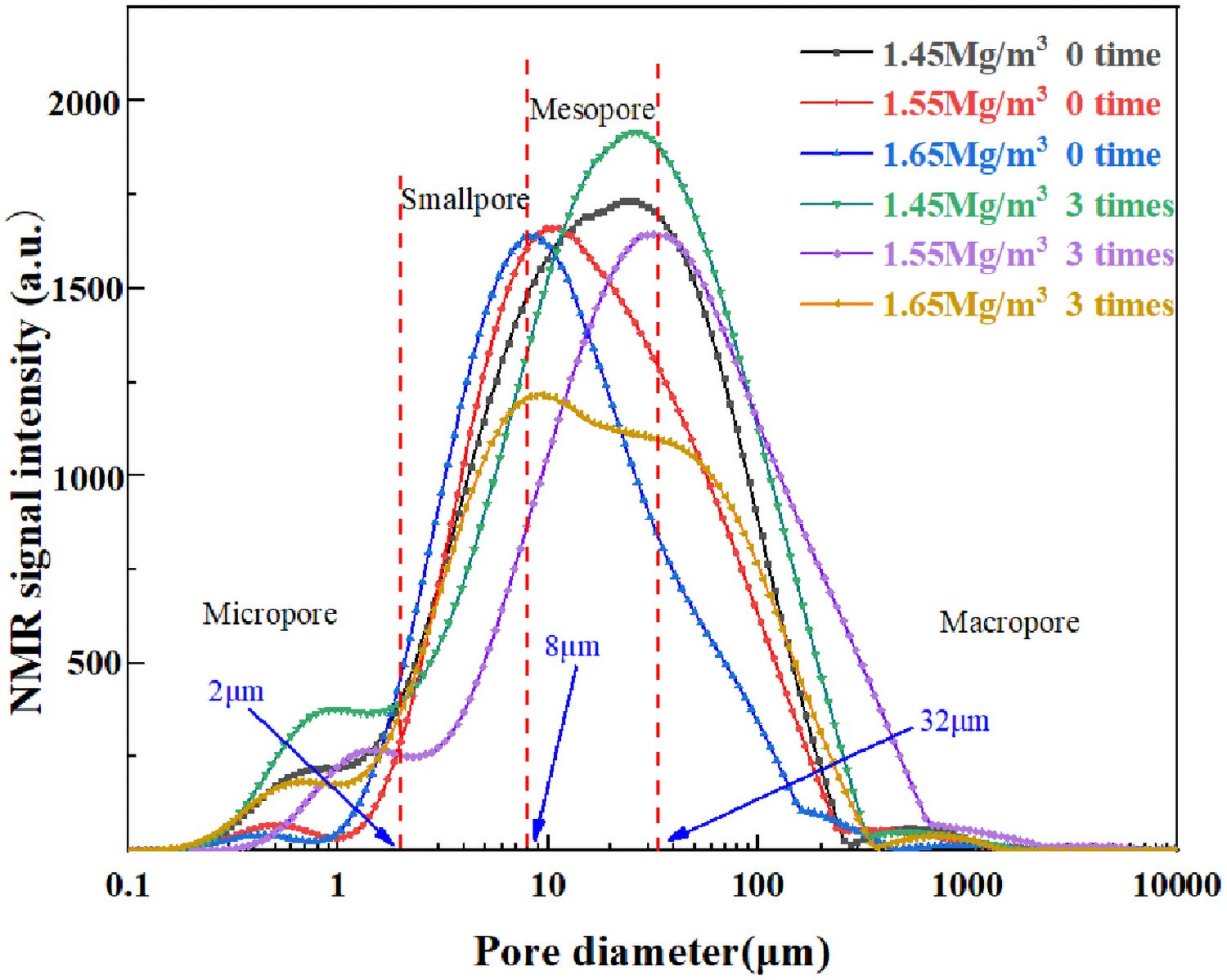
Variations of different pore sizes after different wetting-drying cycles.

NMR signal intensity is proportional to water content, which is related to pore abundance. According to Eq (6), relaxation time T_2_ is linearly proportional to pore size. According to the relaxation time distribution, the pore size distribution of samples with different dry densities was calculated using Eq (9). The *ρ* values are 2.69, 2.51 and, 2.25 um/ms respectively. Therefore, the NMR signal and the pore size distribution signal are established (Fig 11). Results showed that the trimodal pore distribution of all samples primarily consists of a pore diameter range from 0.115 to 2213.5 μm. With increasing dry density, the Peak 1 maximum values are 0.754 μm, 0.493 μm, and 0.374 μm for dry density of 1.45 g/cm^3^, 1.55 g/cm^3^, and 1.65 g/cm^3^ respectively. The peak 2 maximum values for dry densities of 1.45 g/cm^3^, 1.55 g/cm^3^and 1.65 g/cm^3^ are 22.62 μm, 10.55 μm, and 8.21 μm, respectively. However, at Peak 3, the peaks of the three dry densities are all around 552.03μm, proving that with increasing dry density, the predominant micropores and mesopores in the compacted loess gradually decrease, and the influence on macropores is small. With increasing the wetting-drying cycles, the three peak points of 1.45 g/cm^3^ dry density loess increase from 0.754 μm, 22.62 μm and 552.03 μm to 0.693 μm, 25.16 μm and 552.09 μm, respectively. When the dry density of the compressed loess is 1.55 g/cm^3^, the peak points increase from 0.493 μm, 10.55 μm, and 552.03 μm to 1.41 μm, 31.77 μm, and 781.45 μm after three wetting-drying cycles. For 1.65 g/cm^3^ dry density loess, the peak points increase from 0.374 μm, 8.21 μm, and 552.03 μm to 0.613 μm, 9.38 μm, and 679.86 μm, respectively. Therefore, after three wetting-drying cycles, the optimal pore diameter of macropores, mesopores, and micropores in the compacted loess under different dry densities all increased.

The pores in all loess samples were classified into four different grades based on the diameters, with micropores having a diameter from 0 to 2 μm, small pores from 2 to 8 μm, mesopores from 8 to 32 μm, and macropores larger than 32 μm [24, 25]. Dividing the cumulative NMR signal with different diameter ranges by the total NMR signal gives the volume ratio under different pore types (Table 3). Mesopores and macropores are the primary two types of pores in loess samples. Mesopores and macropores in compacted loess with dry densities of 1.45 g/cm^3^ and 1.55 g/cm^3^ accounted for 70% of the total pore volume, and the mesopores and macropores of compacted loess with a dry density of 1.65 g/cm^3^ accounted for 65% of the total pore volume. With increasing dry density, the amount of macropores in the compacted loess rapidly decrease, mesopores and small pores increase, and the change in micropores is relatively small. As the number of wetting-drying cycles increases, the volume occupied by the micropores gradually increases because some fine particles partially flow out during seepage, resulting in more micropores. The decrease in small pores and mesopores is due to the disappearance of the soluble salt between the compacted loess particles during wetting-drying, which causes more overhead structures between the loess particles, leading to an increasing amount of macropores.

**Table 3:** Volume ratio of different pores type in loess samples after wetting-drying cycles.

Dry density (g/cm^3^)	Wetting-drying cycles	Volume ratio of Micropores (%)	Volume ratio of Small pores (%)	Volume ratio of Mesopore (%)	Volume ratio of Macropore (%)
1.45	0	7.05%	22.35%	36.11%	34.49%
1.55	0	2.77%	25.88%	39.66%	31.71%
1.65	0	3.09%	31.83%	41.51%	23.58%
1.45	3	9.69%	18.31%	35.70%	36.30%
1.55	3	8.17%	20.77%	36.70%	34.36%
1.65	3	7.12%	23.42%	32.02%	37.44%

### Reasons for the change of permeability characteristics

Based on the above results, we conclude that as the dry density increases, the compacted loess surface became denser, the saturation permeability coefficient and saturation infiltration rate decreased. However, the wetting-drying cycle presented the opposite result. This is because the amount of aggregates larger than 50 μm gradually decrease with increasing wetting-drying cycles, and the number of aggregates smaller than 10 μm gradually increase [26]. As a result, more tiny aggregates are present in the compacted loess. During infiltration, tiny aggregates will move with the water, and some of them will be removed from the loess structure by the solution, and the connection between the aggregates will gradually change, further leading to the development of a loess overhead structure. In the structure of compacted loess, the total porosity increases with increasing the number of wetting-drying cycles, and the proportion of the total pore volume occupied by macropores increases significantly, leading to the formation of more water channels during infiltration and thus leading to a change in permeability characteristics of compacted loess. With increasing dry density, more tiny aggregates will be produced within the loess aggregates, and the total pore volume decreases, leading to decreasing saturation permeability coefficient and saturation infiltration rate. Therefore, the change in permeability characteristics of compacted loess is determined by aggregate size, loess surface morphology, and the total pore volume occupied by macropores.

## Conclusions

This study explored the permeability characteristics and microstructure changes of compacted loess under different dry densities and wetting-drying cycles. The conclusions drawn from this study are summarized as follows:

1. For compacted loess with the same dry density, as the number of wetting-drying cycles increases, saturation permeability coefficient, saturated infiltration rate, and the initial infiltration rate increases. However, with the same number of wetting-drying cycles, the permeability characteristics of compacted loess show opposite results.
2. With increasing dry density, the proportion of the total area occupied by the aggregates with diameters greater than 10 μm gradually increases, and the changes in aggregates with diameters less than 10 μm were the opposite. Nevertheless, as the number of wetting-drying cycles increases, the total area of aggregates larger than 50 μm gradually decreases, and the total area of aggregates smaller than 10 μm gradually increases.
3. NMR analyses indicate that with increasing dry density, the volume of macropores in the compacted loess rapidly decrease, the volume of mesopores and small pores increase, and the change in micropores is relatively small. Simultaneously, the pore volume of compacted loess with different dry densities increases by 8.56%, 8.61% and 6.15%, respectively.
4. The aggregate size, loess surface morphology, and the total pore volume occupied by macropores. are the main factors that determine the permeability characteristics of compacted loess.

## Supporting information

S1 Dataset The data of X-ray diffraction (XRD) experiments. (XLSX)

S2 Dataset Permeability characteristics test data. (XLSX)

S3 Dataset NMR experimental data. (XLSX)
